# Atypical Presentation of Carbon Monoxide Poisoning With Aphasia

**DOI:** 10.7759/cureus.37019

**Published:** 2023-04-01

**Authors:** Griffin Shimp, Adam Fratczak, Jeffrey A Nielson, Rebecca Perry

**Affiliations:** 1 Emergency Medicine, West Virginia School of Osteopathic Medicine, Lewisburg, USA; 2 Emergency Medicine, Kettering Health, Dayton, USA

**Keywords:** expressive aphasia, oxygen therapy, emergency medicine, altered mental state, focal neurologic deficits, accidental poisoning, applied toxicology, carbon monoxide

## Abstract

We present a patient with carbon monoxide poisoning with a single focal neurological deficit. The patient was found by emergency medical services (EMS) to be resting in his truck with a generator running nearby. On arrival, the patient was hemodynamically stable. The patient was aphasic but did not exhibit any other focal or lateralizing deficits. He was able to communicate by writing clearly and coherently on a sheet of paper. His initial carboxyhemoglobin was 29%, confirming the diagnosis of carbon monoxide poisoning. He was treated with 100% O_2_ via a non-rebreather mask and regained his speech during his ED (emergency department) course. The patient was ultimately hospitalized for continued oxygen treatment and serial examinations. This case highlights the varied presenting symptoms of carbon monoxide poisoning as well as the importance of including a broad differential diagnosis while working up patients with a focal neurologic deficit.

## Introduction

Carbon monoxide (CO) is an odorless, colorless, tasteless gas, and toxicity can result in severe tissue hypoxia. CO poisoning is one of the most common poisonings in the United States, and there are over 1,000 deaths per year secondary to CO [1,2]. Historically, vehicular emissions were the most common source of CO; however, in recent years, other sources have become more prevalent, such as portable heaters and generators [3]. The signs and symptoms of CO poisoning can be broad and nonspecific. These range considerably from headache and simple drowsiness to vision changes, altered mental status, cardiac arrest, non-cardiogenic pulmonary edema, renal failure, rhabdomyolysis, and others. The nonspecific presentation leads to a broad differential diagnosis, including but not limited to intracranial pathologies, migraine, cyanide toxicity, and encephalopathy.

A detailed history is important in the timely diagnosis and treatment of CO poisoning. In order to include CO poisoning in a differential, histories from family members, bystanders, and emergency medical services (EMS) are all valuable. Key historical points such as house fires, multiple victims with similar symptoms, suicide attempts, or proximity to CO-emitting sources should raise CO poisoning suspicion. Treatment that depends on poisoning severity ranges from 100% oxygen to hyperbaric oxygen therapy.

## Case presentation

A 49-year-old male presented to the emergency department (ED) via EMS with a chief complaint of altered mental status. Per EMS, the patient was found confused in his truck with a generator running nearby. EMS reported that the patient was initially nonverbal. EMS was concerned about CO poisoning, and he was subsequently placed on a 15 L non-rebreather en route. On arrival at the ED, the patient had pure expressive Broca aphasia. The patient was able to follow commands, and a full neurological exam was performed, which did not reveal any additional deficits. Interestingly, the patient was able to communicate clearly by writing in full sentences with a pen.

On arrival, the patient’s vital signs revealed a blood pressure of 120/90 mmHg, heart rate of 107 beats/minute, respiratory rate of 20 breaths/minute, oxygen saturation of 100% on room air, and a temperature of 98.7°F. Initial venous blood gas showed a carboxyhemoglobin of 29% (normal: <10%). He remained on the non-rebreather mask at a rate of 15 L. Other lab values, including a complete blood count and basic metabolic panel, were reassuring. Non-contrast computerized tomography of the head did not show any acute intracranial pathology. Although the history and workup were consistent with CO poisoning, an acute stroke could not be entirely ruled out. As a result, computerized tomography angiography of the head and neck was also obtained but did not show any evidence of vascular abnormalities such as stenosis or occlusion. Neurology was consulted, and admission for further evaluation was recommended.

**Figure 1:**
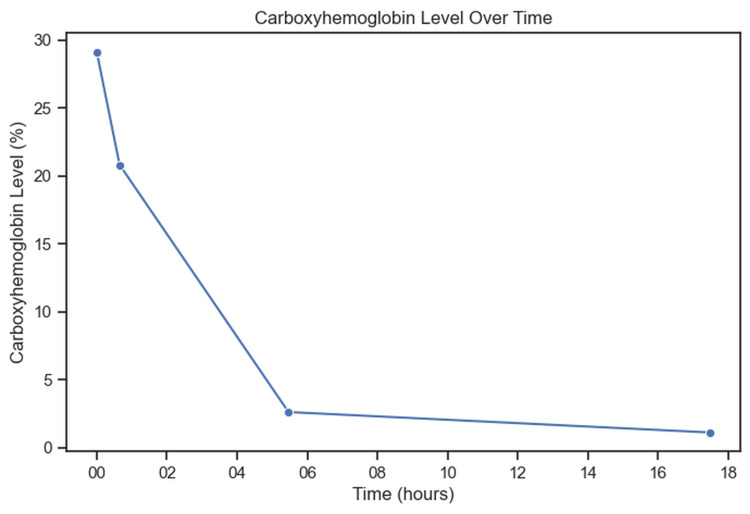
The patient's carboxyhemoglobin level over time

On reevaluation of the patient, he had begun speaking in full sentences. He stated that he was using an electric heater in his truck the previous night and had a generator running outside the vehicle. The patient met the criteria for hyperbaric oxygen treatment and was offered a transfer to an outside facility; however, after shared decision-making, the patient was admitted to the current hospital. A repeat venous blood gas showed a carboxyhemoglobin of 20.8% after approximately 45 minutes of the non-rebreather oxygen therapy. The patient remained stable in the ED and was admitted to the floor for further oxygen therapy and further evaluation. Figure 1 shows the patient's carboxyhemoglobin level over time.

The patient continued on oxygen therapy with eventual normalization of his carboxyhemoglobin levels. Additionally, the likelihood of focal seizure was deemed to be low, and the electroencephalogram was deferred. The patient remained at his neurological baseline and was discharged after one day without further imaging.

## Discussion

CO poisoning affects roughly 50,000 people annually, with 1,200-1,300 deaths per year. Roughly two-thirds of these cases are due to intentional CO exposure, and the remaining cases are secondary to accidental, non-fire causes [2]. These non-intentional cases show a seasonal pattern, with a higher incidence during the winter months [4].

CO has roughly 240 times the affinity for hemoglobin compared to oxygen, resulting in carboxyhemoglobin (COHb) formation. As carboxyhemoglobin levels increase, it creates a leftward shift of the oxygen-dissociation curve, resulting in decreased oxygen release and tissue hypoxia [5]. Furthermore, CO binds to myoglobin leading to cardiac ischemia and decreased cardiac output. CO also binds to mitochondrial cytochrome C oxidase, thereby disrupting oxidative phosphorylation and perpetuating tissue hypoxia [5]. Lastly, CO inhibits cytochrome p450 in the brain, which may contribute to lipid peroxidation and neurologic injury [5].

As mentioned earlier, the signs and symptoms of CO poisoning are variable and often nonspecific, and the correlation between disease severity and COHb level is poor [2]. Presenting symptoms may include headache, dizziness, fatigue, nausea, vomiting, chest pain, shortness of breath, altered mental status, focal neurological deficits, seizures, and coma. The classic “cherry red” skin is rare, nonspecific, and often found postmortem [6]. Interestingly, a 2019 series of 72 patients showed only eight patients with abnormalities on the initial neurological exam, which was lower than the classic description [7]. A prospective cohort study of 230 patients by Henry et al. showed that 37% of hospitalized patients with moderate to severe CO poisoning had evidence of myocardial injury [8].

Diagnosis of CO poisoning is made from an elevated COHb level. It is important to note that a COHb level of less than 3% is normal in nonsmokers and less than 10% in smokers [9]. A level greater than 10% is consistent with CO poisoning. It is also important to note that standard pulse oximetry will be normal in CO poisoning since COHb is interpreted by the sensor as saturated hemoglobin, which was the case in our patient. So, co-oximetry that measures total hemoglobin as well as oxyhemoglobin, methemoglobin, and COHb saturation is the only accurate measurement tool. Routine blood gas analyzers do not include COHb saturation. Additionally, venous blood gas with co-oximetry is sufficient, given that COHb is present in both arterial and venous samples. Further diagnostics such as complete blood count, basic metabolic panel, venous blood gas, electrocardiogram, and chest x-ray should be included in the standard workup.

Initial management is 100% oxygen via a nonrebreathing mask. Indications for hyperbaric oxygen therapy include COHb greater than 25%, COHb greater than 15% in pregnant patients, loss of consciousness, severe metabolic acidosis (pH < 7.25), or evidence of end-organ ischemia. In this case, the patient had a pH of 7.36, not meeting the criteria for hyperbaric oxygen therapy, but the initial COHb was over 25% (29%). As there is some debate regarding the appropriateness of hyperbaric oxygen in the asymptomatic patient with a normal workup, it is not out of the ordinary for a patient of this type to be managed without hyperbaric therapy as this one was.

This case is unique because the presenting symptom of CO poisoning was aphasia. Despite a relatively high COHb saturation, the patient did not exhibit any classic symptoms such as headache, nausea, vomiting, or drowsiness. His focal neurological abnormalities are not the norm in CO poisoning. Maintaining a broad differential diagnosis is an important skill in emergency medicine. This case highlights the importance of expanding the differential for neurologic findings beyond intracranial pathologies. It also illustrates the importance of prehospital medicine and EMS interventions en route to the ED.

## Conclusions

Aphasia was the presenting symptom of CO poisoning in this case, thus making it unique. Despite having a relatively high COHb saturation, the patient showed no classic symptoms such as headache, nausea, vomiting, or drowsiness. Maintaining a broad differential diagnosis is an important skill in emergency medicine. This case emphasizes the importance of expanding the differential diagnosis for neurologic findings beyond intracranial pathologies. It also illustrates the necessity of prehospital medicine and EMS interventions on the way to the ED.
